# Are the urology operating room personnel aware about the ionizing radiation?

**DOI:** 10.1590/S1677-5538.IBJU.2014.0351

**Published:** 2015

**Authors:** Adem Tok, Alparslan Akbas, Nimet Aytan, Tamer Aliskan, Izzet Cicekbilek, Mehmet Kaba, Abdulkadir Tepeler

**Affiliations:** 1 Department of Urology, Faculty of Medicine, Bulent Ecevit University, Zonguldak, Turkey; 2 Department of Urology, Faculty of Medicine, Canakkale 18 Mart University, Canakkale, Turkey; 3 Department of Urology, Faculty of Medicine, Bezmialem Vakif University, Istanbul, Turkey; 4 Department of Urology, Faculty of Medicine, Yuzuncu Yil University, Van, Turkey

**Keywords:** Radiation Injuries, Minimally Invasive Surgical Procedures

## Abstract

**Purpose:**

We assessed and evaluated attitudes and knowledge regarding ionizing radiation of urology surgery room staff.

**Materials and Methods:**

A questionnaire was sent by e-mail to urology surgery room personnel in Turkey, between June and August 2013. The questionnaire included demographic questions and questions regarding radiation exposure and protection.

**Results:**

In total, 127 questionnaires were answered. Of them, 62 (48.8%) were nurses, 51 (40.2%) were other personnel, and 14 (11%) were radiological technicians. In total, 113 (89%) participants had some knowledge of radiation, but only 56 (44.1%) had received specific education or training regarding the harmful effects of radiation. In total, 92 (72.4%) participants indicated that they used a lead apron and a thyroid shield. In the subgroup that had received education about the harmful effects of radiation, the use ratio for all protective procedures was 21.4% (n=12); this ratio was only 2.8% (n=2) for those with no specific training; the difference was statistically significant (p=0.004). Regarding dosimeters, the use rates were 100% for radiology technicians, 46.8% for nurses, and 31.4% for other hospital personnel; these differences were statistically significant (p<0.001). No significant relationship between working period in the surgery room, number of daily fluoroscopy procedures, education, task, and use of radiation protection measures was found.

**Conclusions:**

It is clear that operating room-allied health personnel exposed to radiation do not have sufficient knowledge of ionizing radiation and they do not take sufficient protective measures.

## INTRODUCTION

Recently, the use of open surgical interventions has decreased notably, due to technological developments, improvements, and an increase in the use of minimally invasive methods. Indeed, it has been reported at major medical centers that the ratio of open surgical interventions for urinary system stones is now as low as 1-4% (1). Shock wave lithotripsy, percutaneous nephrolithotomy, endoscopic ureter stone treatments, and retrograde intrarenal operations are frequently used minimally invasive treatment methods for ureter and kidney stones.

During these minimal invasive methods, imaging techniques (e.g. fluoroscopy, ultrasonography, computed tomography) are usually used as guidance. The most commonly used technique is fluoroscopy. However, a major disadvantage of fluoroscopy use is radiation exposure of the patient, surgeon, and other operating room personnel. As a result of this exposure to radiation, deterministic and stochastic effects (mutations and carcinogenesis) occur in the body (2). For this reason, the International Radiation Commission recommends not exceeding 20 mSv/year during a 5-year occupational period (3). The effects of ionizing radiation change depending on the radiation dose, duration, and whether and how much protection is used.

Preventive measures should be taken to protect against the effects of radiation. The most important include use of lead aprons, thyroid shields, and radiation-protective gloves and eyeglasses. Furthermore, it is important to use dosimeters to determine the cumulative amount of radiation exposure. With such information, it can be determined which subjects do not have enough protection against exposure to radiation (4). Attitudes and behavior of endourologists and other team members exposed to radiation during surgiry were evaluated by some investigators (4, 5).

Although operating room personnel are an important occupational group with increased risk of radiation exposure, there is no study specifically evaluating their awareness about this risk. In this study, we evaluated attitudes and behavior regarding ionizing radiation of urology operating room personnel.

## MATERIALS AND METHODS

With the approval of the local ethics committee, a questionnaire survey was sent by e-mail to 183 operating room personnel (nurses, radiology technicians and other personnel) (age range 20-50 years) working in urology surgery rooms at various state hospitals, private hospitals, training research hospitals, and medical faculty hospitals between June and August 2013. The survey was prepared on the Google Docs™ website. Because of the characteristics of the software, the participants remained anonymous. Participants were informed that the results of the survey would be used for scientific purposes only and that their identities would not be determined or recorded.

The questionnaire included 13 questions. These concerned demographic informations, such as job, age, educational background, work duration in surgery rooms, and number of daily endourological cases and how many used fluoroscopy, whether a dosimeter was used, and, if so, whether periodic exposure measurements were made, whether the participant had an understanding of the harmful effects of radiation, whether they had received specific training about the harmful effects of radiation and about protective methods against radiation and which one(s) they used, and whether there was a radiation warning sign in areas where fluoroscopy was used.

The survey was self-administered and was not validated. After gathering the results of the survey, data were analyzed using the SPSS software (ver. 18.0).

## RESULTS

In total, 127 (69.4%) participants completely answered and returned the questionnaire. Of the participants, 62 (48.8%) were nurses, 51 (40.2%) were other operating room personnel, and 14 (11%) were radiology technicians. The average age of responders was calculated as 32.0±5.9 (range 20-50) years. Regarding education, in order, 47 (37%) had a bachelor's degree, 30 (23.6%) had a 2-year associate degree, and 30 (23.6%) had completed high school. Other demographic information is provided in Table-1. Most of the participants were staff at university hospitals (31.5%) and training and research hospitals (40.9%). Most of the personnel indicated that they were involved in two (30.7%) or three (30.7%) endourological surgeries per day. In these cases, fluoroscopy use was typically once (34.6%) or twice (12.6%) per day. Regarding work experience in urology surgery rooms, 16 (12.6%) indicated that they had worked there less than 1 year, 70 (55.1%) between 1 and 5 years, 24 (18.9%) between 5 and 10 years, and 17 (13.4%) for more than 10 years. No significant relationship was found between period of working in the surgery room, number of daily fluoroscopy procedures, education, task, and use of protection from radiation.

**Table 1:** Demographic characteristics of participants.

Questions	Answers	n	(%)
Job	Other personnel	51	(40.2%)
	Nurse	62	(48.8%)
	Radiological technician	14	(11%)
Mean age	32.01±5.9 years		
Education	Primary school	19	(15%)
	High school	30	(23.6%)
	2-years associate degree	30	(23.6%)
	Bachelor's degree	47	(37%)
	Postgraduate	1	(0.8%)
For how long (years) have you worked in a urology	<1 year	16	(12.6%)
surgery room?	1-5 years	70	(55.1%)
	6-10 years	24	(18.9%)
	>10 years	17	(13.4%)
Your corporation	Private Hospital	16	(12.6%)
	State Hospital	19	(15%)
	Training and research Hospital	52	(40.9%)
	University Hospital	40	(31.5%)

For surgeries using fuoroscopic imaging, 59 (46.5%) participants indicated that they used dosimeters, and 54 indicated that they gathered monthly and yearly measurements. Although 50% of personnel working at university hospitals and 55.8% at training and research hospitals indicated that they used dosimeters, participants from private hospitals indicated that they did not (p=0.001).

The dosimeter usage rate was 100% for radiology technicians, 46.8% for nurses, and 31.4% for other operating room personnel; these differences were statistically significant (p<0.001). Of the participants, 113 (89%) reported having information about the harmful effects of radiation, but the number of participants who had been specifically educated about these effects was 56 (44.1%); the training rate was 100% for radiology technicians. Of the participants, 92 (72.4%) indicated that they used lead aprons along with thyroid shields. Protective measures are presented in Table-2. In the group who had received education about the harmful effects of radiation, the use ratio for all protective measures combined, that is, lead apron + thyroid shield + eyeglasses +l eaded gloves was 21.4% (n=12), compared to 2.8% (n=2) in the group without specific training; this difference was statistically significant (p=0.004). Of the 14 personnel who reported using all four protective measures, 11 were nurses.

**Table 2:** Other questionnaire responses by participants.

Questions	Answers		n (%)
What is the average number of endourology cases you attend daily?	1	7	(5.5%)
	2	39	(30.7%)
	3	39	(30.7%)
	4	18	(14.2%)
	>4	24	(18.9%)
Of the daily operations you attend, how many involve fluoroscopy?	1	44	(34.6%)
	2	40	(31.5%)
	3	27	(21.3%)
	4	9	(7.1%)
	>4	7	(5.5%)
Do you have dosimeter?	Yes	59	(46.5%)
	No	68	(53.5%)
Do you have monthly and yearly measurements from your dosimeter?	Yes	54	(42.5%)
	No	73	(57.5%)
Do you have an understanding of the harmful effects of radiation?	Yes	113	(89%)
	No	14	(11%)
Did you take specific training about the harmful effects of radiation?	Yes	56	(44.1%)
	No	71	(55.9%)
Which protective methods do you use against the effects of radiation?	Lead apron	13	(10.2%)
	Thyroid shield	4	(3.1%)
	Apron-Thyroid shield	92	(72.4%)
	Apron-Gloves	1	(0.8%)
	Apron-Thyroid shield-Gloves	1	(0.8%)
	Apron-Thyroid shield-Eyeglasses	2	(1.6%)
	Apron-Thyroid shield-Gloves-Eyeglasses	14	(11.0%)
Is there a radiation warning sign in the area(s) where fluoroscopy is used?	Ye s	62	(48.8%)
	No	65	(51.2%)

Of the participants, 65 (51.2%) indicated that there was no warning sign in areas where radiation was used. Moreover, 25 (40.3%) participants who indicated that warning signs were present worked at training and research hospitals (p=0.005).

## DISCUSSION

Ionizing radiation to which staff members are exposed during medical diagnostic interventions and treatments is a health issue, and the harmful effects of radiation must be taken into consideration. Particularly, in recent years, because of the increasing number of endourological interventions, urologists have a key role in controlling exposure to ionizing radiation for themselves, other health personnel, and, indeed, their patients (6). The deterministic effects of ionizing radiation-that is, cell death and ultimately organ dysfunction in sufficiently large doses-are rarely encountered, even in those working with radiation. However, long-term exposure to low doses that do not cause immediate cell damage can modify cells and result in stochastic effects (mutations and carcinogenesis). To minimize these effects, “ALARA” (as low as reasonably achievable) principles must be followed (7).

‘It has been shown in many studies that most urologists are poorly aware of the radiation exposure to themselves, and their patients, and that insufficient precautions are commonly taken against radiation (4, 8, 9). Indeed, it has been reported that urologists and their assistants are exposed to considerably greater levels of radiation than are other operating room personnel (10). Although the dose of radiation exposure per case that personnel receive is lower than the level that surgeons receive, the cumulative level of radiation exposure may be higher for operating room personnel. Although statistically insignificant, nurses gave more importance to protective measures (11). This fact probably was due to nurses work closer to the radiation source than the radiology technicians and other personnel. In our study, although fluoroscopy was used commonly in urological procedures and the harmful effects of radiation are known generally by all personnel exposed, insufficient precautions were taken.

The use of appropriate protective equipment greatly reduces the harmful effects of ionizing radiation (6, 12). However, studies have shown that lead aprons, thyroid shields, leaded gloves, and eyeglasses are not commonly used in combination (4, 6). Also in this study, most of the participants who used protection used aprons and thyroid shields; the usage of gloves and eyeglasses was rare. The ratio of the combined use of apron + thyroid shield + eyeglasses + leaded gloves in personnel educated regarding radiation was 21.4%, compared to 2.8% in personnel without specific education (p=0.004). Training regarding the harmful effects of radiation can substantially increase the use of protective measures. Söylemez et al. reported that because most protective equipment was inappropriate ergonomically, it was little used (13).

According to Turkish Radiation Safety Regulations about radiation protection, doses cannot exceed the individual dose limits (14). Moreover, if the yearly dose could exceed 30% of the permitted level, personal dosimeters must be used. In this study, 46.5% of the participants (59 persons) used dosimeters; of them, 49 were from university and training and research hospitals. The less frequent use of dosimeters in state and private hospitals may be due to the less common performance of fluoroscopy or inadequate education about radiation and its affects in these institutions.

Although most of the participants (89%) in the survey reported some knowledge of the harmful effects of radiation, only 44.1% of them had received specific education regarding the harmful effects of radiation. Using inadequate protection against radiation is a result of inadequate education concerning the issue (4, 6). Radiation technicians attended endourological surgeries as part of their undergraduate and associate degree programs, with the expected results, presumably due to their education.

The use of appropriate warning signs in radiation areas is important for patients, relatives, and other health personnel working in these areas. Although warnings were displayed in many surgery rooms, inadequate care and insufficient preventative measures were being taken when entering such areas. Most of the participants indicated that they were involved in surgeries using fluoroscopy at least once or twice per day. However, despite the increase in the number of fluoroscopy procedures, there has been no corresponding change in the radiation protection methods used by surgery room personnel. Unwanted side effects of radiation exposure may be seen in surgery room personnel over the long term.

In the survey, fluoroscopy was performed frequently at university and training and research hospitals. This can be explained by the availability of appropriate equipment at these hospitals, as indicated in other studies (4). Although the physicians and their assistants involved differ among the surgical procedures performed in these centers, the allied health personnel tend to be identical, which causes them to be exposed to ionizing radiation often, if not continuously. However, because the radiation dose decreases with distance from the fluoroscopy source, this may be militate in favor of nurses and other personnel who are not directly involved in the surgery and so are more distant from the radiation.

The main limitation of this study is the small number of responders. Although only a small group of personnel working in different hospitals of Turkey were included in this study, we believe that further studies in larger populations in different age groups will provide more information about this specific topic. The other point is that the responders were young. This may be related to the fact that Internet usage is more common in young age population. Endourology procedures using fluoroscopy guidance have been more popular in recent years. Young urologists and operating room personnel are especially more involved in these procedures. Despite these limitations we believe that this study emphasize the importance of radiation protection for operation room personnel. To the best of our knowledge this is the first study evaluating knowledge and attitude of operation room personnel about radiation exposure in the literature.

## CONCLUSIONS

The application of ALARA principles in areas where fluoroscopy is used is necessary and, indeed, essential for occupational health. However, surgery room personnel who are subjected to radiation exposure did not have sufficient information regarding ionizing radiation and did not take sufficient preventive measures. We consider that this was likely due to insufficient education. It is important that personnel who work with radiation in these departments receive training during their basic education or as a part of in-service training. Beyond this, the use of dosimeters and determination of exposure levels must be enforced by the authorities.

## SURVEY Survey regarding knowledge level of surgery room personnel about ionizing radiation

This form was prepared only to assess the level of knowledge of radiation of surgery room personnel in Turkey. The information obtained from this survey will not be used to criticise and/or accuse any individual or corporation of anything. The objective of this survey is to attract the attention of medical staff to an important issue, namely radiation exposure.

**Table:**

Job		
	Other personnel	( )
	Nurse	( )
	Radiological technician	( )
**Age**		( )
	Education	
	Primary school	( )
	High school	( )
	2-years associate degree	( )
	Bachelor's degree	( )
	Postgraduate	( )
**For how long (years) have you worked in a urology surgery room?**		
	<1 year	( )
	1-5 years	( )
	6-10 years	( )
	>10 years	( )
**Your corporation**		
	Private Hospital	( )
	State Hospital	( )
	Training and research hospitals	( )
	University Hospital	( )
**What is the average number of endourology cases you attend daily?**		
	1	( )
	2	( )
	3	( )
	4	( )
	>4	( )
**Of the daily operations you attend, how many involve fluoroscopy?**		
	1	( )
	2	( )
	3	( )
	4	( )
	>4	( )
**Do you have dosimeter?**		
	Yes	( )
	No	( )
**Do you have monthly and yearly measurements from your dosimeter?**		
	Yes	( )
	No	( )
**Do you have an understanding of the harmful effects of radiation?**		
	Yes	( )
	No	( )
**Did you take specific training about the harmful effects of radiation?**		
	Yes	( )
	No	( )
**Which protective methods do you use against the effects of radiation?**		
	Lead apron	( )
	Thyroid shield	( )
	Gloves	( )
	Eyeglasses	( )
**Is there a radiation warning sign in the area(s) where fluoroscopy is used?**		
	Yes	( )
	No	( )
